# Research Progress of Benzothiazole and Benzoxazole Derivatives in the Discovery of Agricultural Chemicals

**DOI:** 10.3390/ijms241310807

**Published:** 2023-06-28

**Authors:** Yue Zou, Yong Zhang, Xing Liu, Hongyi Song, Qingfeng Cai, Sheng Wang, Chongfen Yi, Jixiang Chen

**Affiliations:** 1 National Key Laboratory of Green Pesticide, Key Laboratory of Green Pesticide and Agricultural Bioengineering, Ministry of Education, Center for R&D of Fine Chemicals of Guizhou University, Guiyang 550025, China; zy15186251853@163.com (Y.Z.); zhangyong001103@163.com (Y.Z.); 15721762850@163.com (X.L.); shy2637649950@163.com (H.S.); qingfengc119@163.com (Q.C.); swang3004@163.com (S.W.); 2 Guizhou Rice Research Institute, Guizhou Academy of Agricultural Sciences, Guiyang 550025, China; 13608581558@163.com

**Keywords:** benzoxazole, benzothiazole, agrochemical, SAR, mechanism

## Abstract

Benzoxazole and benzothiazole have a broad spectrum of agricultural biological activities, such as antibacterial, antiviral, and herbicidal activities, which are important fused heterocyclic scaffold structures in agrochemical discovery. In recent years, great progress has been made in the research of benzoxazoles and benzothiazoles, especially in the development of herbicides and insecticides. With the widespread use of benzoxazoles and benzothiazoles, there may be more new products containing benzoxazoles and benzothiazoles in the future. We systematically reviewed the application of benzoxazoles and benzothiazoles in discovering new agrochemicals in the past two decades and summarized the antibacterial, fungicidal, antiviral, herbicidal, and insecticidal activities of the active compounds. We also discussed the structural–activity relationship and mechanism of the active compounds. This work aims to provide inspiration and ideas for the discovery of new agrochemicals based on benzoxazole and benzothiazole.

## 1. Introduction

In global agricultural production, plant diseases, insects, and weed damage are the main causes of crop yield loss [1,2]. Fungi [3,4], bacteria [5,6,7], plant viruses [8,9], pests [10,11], weeds [12], nematodes [13,14,15,16], and mites [17] cause huge economic losses to the world’s agriculture every year. At present, the use of agrochemicals is still one of the most effective means to control plant diseases, insects, and grass damage, especially in the management of pest resistance and resistant weeds [18,19]. More importantly, when pests (such as armyworms [20], locusts [21], and walkers [22]) break out in large areas, the use of highly efficient chemical pesticides is the most effective strategy for rapid pest control [23]. However, long-term use of traditional agrochemicals will not only pollute the environment but also increase the resistance of pathogens [24], resulting in more difficult management of plant diseases, insects, and weeds [7,25,26]. Therefore, the development of new agrochemicals with unique action mechanisms to replace traditional pesticides is an urgent problem to be solved in the management of plant diseases, pests, and grass diseases.

**Figure 1:**
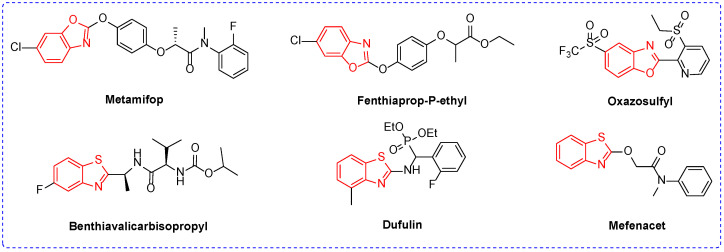
Chemical structure of some pesticides containing benzoxazole or benzothiazole scaffolds.

Benzoxazole is a combination of a benzene ring and an oxazole ring; benzothiazole is the bioisostere of benzoxazole. They are widely used in drug research and development as the core scaffold structure [27,28,29,30,31,32] and play an important role in drug discovery. Twenty years ago, the research on benzothiazole and benzoxazole was widely focused on the field of medicine [33,34,35,36]; on the contrary, there was little research in the field of agrochemicals. However, 10 years ago, there was a large amount of research on benzothiazole and benzoxazole in new agrochemicals. In terms of commercial agrochemicals, benzoxazole and benzothiazole agrochemicals play an important role. For example, the herbicides metamifop (Figure 1) and fenoxaprop-p-ethyl are acetyl-coenzyme A carboxylase inhibitors, which inhibit the growth of grasses mainly by inhibiting the synthesis of plant fatty acids, eventually leading to the death of plants [37,38,39,40]. Mefenacet, a systemic herbicide, is an inhibitor of cell generation and division, which can prevent cell division and elongation in weed meristem and has a good control effect on barnyard grass [41]. The fungicide benthiavalicarbisopropyl has an inhibitory effect on the sporangia formation and germination of Phytophthora at low mass concentrations. The mechanism of action is still unclear, but it does not affect the oxidation and synthesis of nucleic acid and protein [42,43]. The antiviral agent Dufulin has been widely used against tomato virus disease, cucumber virus disease, tobacco virus disease, and southern rice black-streaked dwarf virus disease [44,45,46]. Oxazosulfyl, the first benzoxazole insecticide with a broad spectrum of insecticidal activity, is currently mainly used to control rice pests, but its mechanism of action is still unclear [47,48].

Benzoxazole and benzothiazole have stable structures and are easily modified, which play an important role in the discovery of new agrochemicals. Research on the discovery of new agrochemicals based on benzoxazole and benzothiazole scaffolds may be strengthened in the future. There is no comprehensive review of benzoxazole and benzothiazole derivatives in the discovery of novel agrochemicals. Herein, we summarize the benzoxazole and benzothiazole derivatives in the application of new types of agricultural chemicals, perform analysis of the benzoxazole and benzothiazole compounds in terms of antibacterial, antifungal, antiviral, weeding, and insecticidal activity, and discuss the structure–activity relationship (SAR) and mechanism of action. It is hoped that this review provides new clues and inspiration for the discovery of new benzoxazole and benzothiazole agrochemicals.

## 2. Antibacterial Activity

Diseases caused by plant bacteria have seriously restricted the safe production of crops and caused huge output and economic losses to world agriculture every year [49,50]. However, sustained and effective management of these plant bacterial diseases is extremely difficult and often requires integrated management strategies [51,52,53]. The long-term use of chemical antimicrobials has led to the evolution of resistance in bacteria [54]. This puts forward higher requirements for the development of antimicrobial agents and the management of plant bacterial diseases.

**Figure 2:**
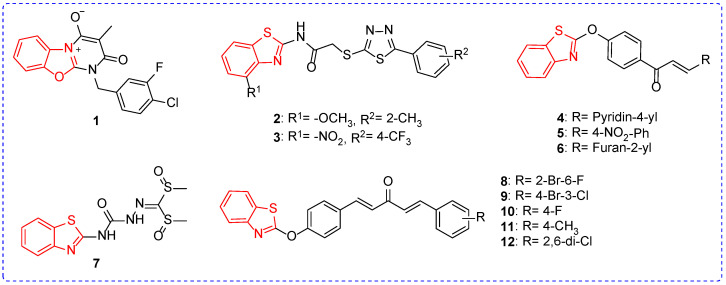
Chemical structure of benzoxazole and benzothiazole antibacterial active compounds **1**–**12**.

Some benzoxazole derivatives or benzothiazole derivatives have good antibacterial activity (Figure 2). For example, the EC_50_ values of compound **1** against *Xanthomonas oryzae pv.oryzicola (Xoc)* and *Xanthomonas citri* subsp. *Citri (Xac)* were 47.6 mg/L (Table 1) and 36.8 mg/L, respectively [55]. In addition, compound **1** showed good antibacterial activity by up-regulating the expression of Succinate dehydrogenase (SDH) during oxidative phosphorylation, thereby inhibiting bacterial reproduction. At a concentration of 100 mg/L, the inhibition rate of compound **2** against *Xanthomonas oryzae pv.oryzae (Xoo)* was 52.4%. Based on compound **2**, the methoxy group was replaced with the nitro group, and the methyl group at position-2 of the benzene ring was replaced with the trifluoromethyl group at position-4 of the benzene ring. The inhibition rate of compound **3** on *Ralstonia solanacearum* (*Rs*) was 71.6% [56]. In addition, the introduction of the pyridine e group increased the broad spectrum of antibacterial compounds. For example, the antibacterial activities of compound **4** against *Xoo*, *Xac*, and *Rs* were 52.40%, 50.97%, and 36.49%, respectively. If the pyridyl group was replaced by the electron-withdrawing group, the antibacterial activity of the compound was enhanced. For example, the EC_50_ value of compound **5** against *Xoo* was 38.97 mg/L, while the EC_50_ value of compound **6** against *Xac* was 13.42 mg/L [57]. The EC_50_ value of compound **7** against *Xoo* was 11.4 mg/L. In addition, compound **7** can not only change cell morphology, but also reduce the pathogenicity of *Xoo* to rice by inhibiting the formation of cell biofilms, thereby affecting cell division [58]. The EC_50_ values of compounds **8** and **9** against *Xoo* were 76.1 and 86.1 mg/L. However, the antibacterial activity of compound **10** (EC_50_ = 20.0 mg/L) was significantly increased when a fluorine atom was introduced into the para position of the benzene ring. In addition, the introduction of para-methyl or ortho-chlorine atoms made the compounds exhibit good antibacterial activity against *Xac*. For example, compounds **11** and **12** had EC_50_ values of 35.7 and 28.5 mg/L for *Xac*. Interestingly, compound **11** can cause fold and damage to cell surface morphology, and the higher the concentration of the compound, the greater the degree of damage on the cell surface [59].

## 3. Antifungal Activity

There are a wide variety of fungal diseases in plants, and their distribution is widespread [60,61]. Fungal diseases not only affect the yield and quality of crops, but also some fungi can secrete toxins and metabolites that are harmful to humans when they infect crops [62,63]. At present, the use of chemical agents is still one of the main methods of fungal disease activity management. In recent years, the research on benzoxazole and benzothiazole fungicidal compounds has made great progress.

**Figure 3:**
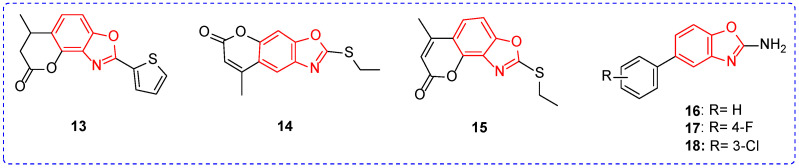
Chemical structure of benzoxazole antifungal active compounds **13**–**18**.

Some benzoxazoles or benzothiazoles have shown excellent fungicidal activity. For example, compound **13** (Figure 3) had an EC_50_ value of 0.3 mg/L (Table 2) for *Alternaria brassicae*, which was superior to the commercial agent carbendazim (EC_50_ = 47.0 mg/L) [64]. At a concentration of 90 mg/L, the protective effect and treatment activities of compounds **14** and **15** against *Botrytis cinerea (B. cinerea)* were greater than 88% [65]. The EC_50_ value of compound **16** for *B. cinerea* was 2.40 mg/L, and the introduction of fluorine or chlorine atoms to the phenyl was conducive to the improvement of fungicidal activity of the compound. For example, compounds **17** and **18** for *B. cinerea* had EC_50_ values of 1.81 and 1.69 mg/L. In addition, compound **16** may show fungicidal activity by binding to the active site of the sec14p target of fungi [66].

**Figure 4:**
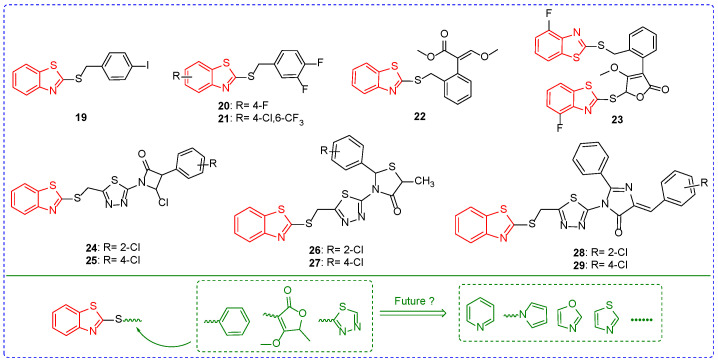
Chemical structures and modified fragments analysis of benzothiazole antifungal active compounds **19**–**29**.

The IC_50_ value of compound **19** (Figure 4) for *B. cinerea* was 1.4 μM (Table 3), and the addition of methylene between benzothiazole and aryl increased the fungicidal activity of the compound [67]. At a concentration of 50 mg/L, the inhibitory rates of compound **20** against *Rhizoctonia solani (R. solani)*, *B. cinerea*, *Dothiorella gregaria (D. gregaria)*, and *Colletotrichum gossypii (C. gossypii)* were 92%, 97%, 89%, and 78%. Moreover, the introduction of chlorine atoms and trifluoromethyl compounds was not beneficial to the fungicidal activity of the compounds. For example, the inhibitory rates of compound **21** against *R. solani*, *B. cinerea*, *D. gregaria*, and *C. gossypii* were 40%, 67%, 35%, and 37% [68]. The EC_90_ values of compound **22** on *Sphaerotheca fuliginea (S. fuliginea)* and *Pseudoperoniospora cubensis (P. cubensis)* were 6.17 and 46.32 mg/L, respectively [69]. The inhibition rates of compound **23** on *S. fuliginea* and *P. cubensis* were 67% [70] because the introduction of large steric groups reduced the fungicidal activity of the compound. Compounds **24**, **26**, and **28** showed inhibition rates of 69%, 55%, and 65% against *Phytophthora infestans (P. infestans)* at concentrations of 100 ppm. The fungicidal activities of compounds **24**, **26,** and **28** were reduced when chlorine atoms on the position-2 of the benzene ring were replaced by position-4 fluorine atoms of the benzene ring. For example, compounds **25**, **27**, and **29** have inhibition rates against *P. infestans* of 58%, 53%, and 58% [71]. 

The position-2 of benzothiazoles replaced by thioether is a good fungicidal scaffold structure, which has the value of further optimization and derivation. Currently, the framework is mainly combined with benzene, furanone, and thiadiazole. In the future, it may be considered to introduce thiazole, oxazole, and pyridine on sulfur atoms to optimize the structure.

**Figure 5:**
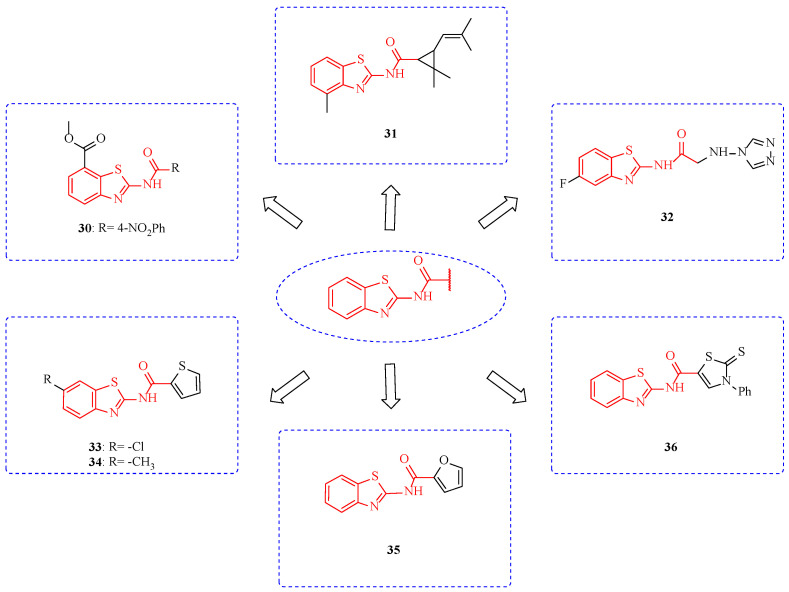
Chemical structures of benzothiazole fungicidal active compounds **30**–**36**.

Amide bonds can form hydrogen bonds with target proteins, and compounds obtained by an organic combination of benzothiazole and amide often show good fungicidal activity [72]. At the concentration of 1000 mg/L, compound **30** (Figure 5) showed an inhibition rate of 88.9% (Table 4) against *B. cinereal*—the 4-nitrophenyl group was beneficial to improve the fungicidal activity of the compound. Interestingly, compound **30** showed better fungicidal activity in vivo than in vitro, suggesting that compound **30** may enhance plant disease resistance [73]. At a concentration of 50 mg/L, the inhibition rates of compound **31** on *B. cinerea* and *Gibberella zeae (G. zeae)* were 80% and 75%, respectively, suggesting that the introduction of permethric acid had no significant contribution to the fungicidal activity of the compound [74]. The EC_50_ values of Compound **32** against *Ustilago tritici*, *Puccinia striiformis*, *Puccinia triticina*, *Blumeria graminis*, *Dickeya oryzae*, and *Ustilag ohordei* are were all less than 0.8 mmol/L [75]. The inhibition rates of compounds **33** and **34** against *Helminthosporium maydis* were 78.6% and 80.6%. The fungicidal activity of the compound was not significantly improved by the introduction of electron-donating or electron-absorbing groups at position-6 of the benzothiazole ring. This suggests that the fungicidal activity of the compound in this structure is independent of the electron density at position-6 of the benzothiazole ring. In the future, spatial effects, hydrogen bonding, and water transport may be considered [76]. When thiazoles in the structure of compounds **33** and **34** were replaced with oxazoles, the fungicidal activity and broad spectrum of the compounds increased. For example, compound **35** had inhibition rates of 93.8%, 94.1%, 93.4%, 94.6%, and 94.5% against *R. solani*, *B. cinereal, G. zeae*, *Helminthosporium maydis*, and *Sclerotinia sclerotiorum (S. sclerotiorum)* [77]. Compound **36** showed a certain inhibitory effect on *Fusarium oxysporum (F. oxysporum)* (MIC 12.5 mg/mL) [78].

**Figure 6:**
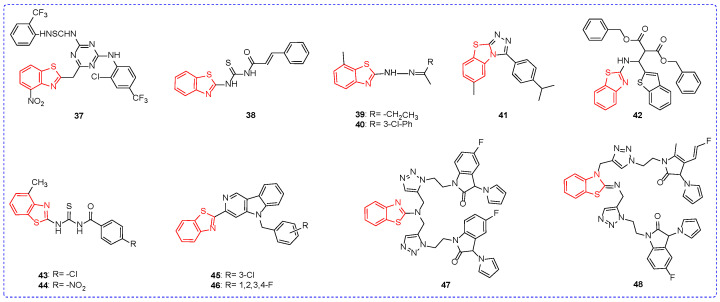
Chemical structures of benzothiazole fungicidal active compounds **37**–**48**.

At the concentration of 100 mg/L, compound **37** (Figure 6) had inhibition rates of 38% (Table 5) to *Alternaria alternata* and 39% to *Aspergillus niger*, respectively. In addition, compound **37** may show fungicidal activity by inhibiting spore germination [79]. Under the condition of concentration of 250 mg/L, compound **38** *G. zeae* inhibition rate was 53.5% [80]. At the concentration of 100 mg/L, the inhibition rate of compound **39** against *Sclerotinia sclerotiorum* was 87.5%. However, the substitution of the alkyl group with the aromatic ring is not conducive to the fungicidal activity of the compound, for example, compound **40** showed 43.8% inhibition of *S. sclerotiorum* [81]. Under the condition of 50 mg/L, the inhibition rate of compound **41** to *R. solani* was 70.43% [82]. The inhibition rate of compound **42** against *F. oxysporum* was 60.53% [83]. At the concentration of 10 mg/L, the average inhibitory zone diameter of compound **43** against *Aspergillus oryzae (A. oryzae)* was 0.81 mm. However, the replacement of chlorine atoms with nitro groups had no significant effect on the fungicidal activity of compounds; for example, the average diameter of the inhibition zone of compound **44** against *A. oryzae* was 0.81 mm [84]. At the concentration of 50 mg/L, the inhibitory activities of compounds **45** and **46** against *Rape sclerotinia rot* were 80.08% and 81.61%, respectively [85]. The ED_50_ values of compounds **47** and **48** for *R. solani* are 0.96 µM and 1.48 µM, respectively, which may be due to amines having stronger alkalinity than imines. In addition, compound **48** binds to the CYP51 site of fungi, hindering the synthesis of fungal cell membranes and, thus, inhibiting the normal growth of fungi [86].

## 4. Antiviral Activity

**Figure 7:**
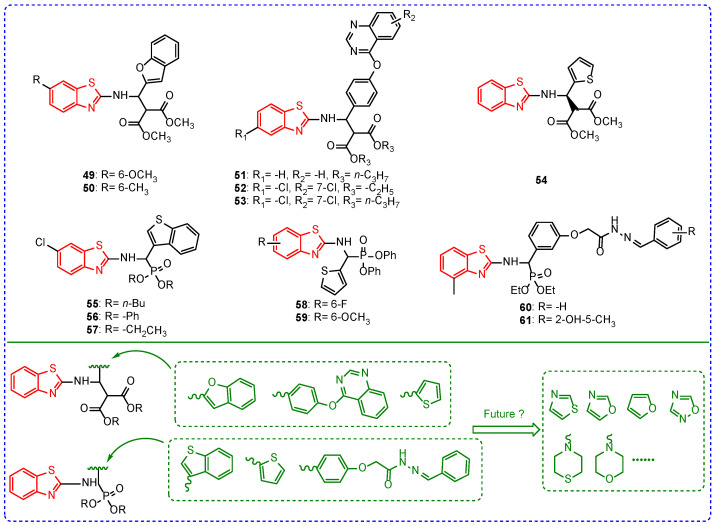
Chemical structures and modified fragments analysis of benzothiazole antiviral active compounds **49**–**61**.

Effective management of plant viral diseases has been one of the hotspots in the field of plant protection [87,88,89]. Plants do not have a complete immune metabolism system, and, once the virus invades the plant, it will reproduce indefinitely in the plant until the plant dies [90,91]. Therefore, plant viral diseases are more difficult to manage than bacterial diseases, fungal diseases, pests, and weeds [92,93,94]. Many studies have been conducted on benzothiazoles against plant virus diseases; some have good antiviral activities. For example, at the concentration of 500 mg/L, the treatment activities of compounds **49** and **50** (Figure 7) against tobacco mosaic virus (TMV) were 52.23% and 54.41% (Table 6), respectively [95]. The electron-donating group in the benzothiazole ring may be an important factor for the antiviral activity of compounds **49** and **50**. The protective activity of compound **51** against TMV was 39.27%. In addition, the introduction of chlorine atoms increased the antiviral activity of the compound; for example, the protective activities of compounds **52** and **53** against TMV were 55.96% and 54.21% [96]. The inhibition rate of compound **54** against TMV was 28.2%, while its racemic activity against TMV was 35.4% [97]. Compounds **55** and **56** had treatment activities against TMV of 37.9% and 35.8%. When the alkyl part of the amino phosphonate of these compounds was ethyl, the compounds showed better antiviral activity. For example, the treatment activity of compound **57** against TMV was 48.1% [98]. The treatment activity of compound **58** against TMV was 48.2%. Replacing the fluorine atom of compound **59** with a methoxy group had no significant effect on the antiviral activity of the compound. For example, the treatment activity of compound **59** against TMV was 47.2% [99]. The treatment, protection, and passivation of compound **60** against TMV were 33.2%, 65.1%, and 45.7%, while, for compound **61** against TMV, they were 74.3%, 78.7%, and 94.3%. Molecular docking found that benzothiazole rings are important for the antiviral activity of these compounds, and the hydrazone’s structure can affect the compounds’ antiviral activity [100]. The combination of benzothiazoles with diesters or amino phosphonate had good antiviral activity, which showed the advantage of the skeleton structure in antiviral activity. Currently, benzothiazole, thiazole, benzothiophene, and benzofuran structures are mainly introduced into benzothiazole scaffolds. In the future, the introduction of thiazole, oxazole, and morpholine rings may be considered to find molecules with higher antiviral activity.

**Figure 8:**
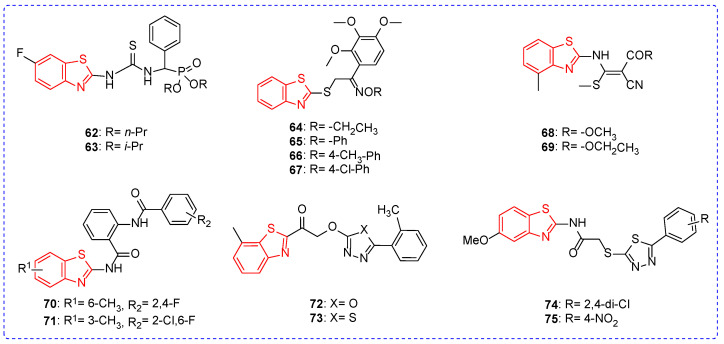
Chemical structures analysis of benzothiazole antiviral active compounds **62**–**75**.

At a concentration of 500 mg/L, the treatment activity of compound **62** (Figure 8) against TMV was 52.9% (Table 7), and the replacement of straight-chain alkanes with branched-chain alkanes resulted in a decrease in the antiviral activity of the compound; for example, compound **63** had a treatment activity against TMV of 46.6% [101]. The substitution of alkyl of compound **64** (30.9%) with benzene ring was beneficial to the improvement of the anti-TMV activity of compound **64** (30.9%). For example, compounds **65**, **66**, and **67** had anti-TMV activities of 32.1%, 38.1%, and 44.0%, respectively, at a concentration of 0.05% [102]. Under the condition of concentration of 50 mg/L, the inhibition rate of compound **68** against Cucumber mosaic virus (CMV) was 46.3%, while the growth of the alkyl chain had little effect on the antiviral activity of the compound; for example, the inhibition rate of compound **69** against CMV was 45.1% [103]. At the concentration of 500 mg/L, the inhibition rate of compound **70** on TMV was 44.5%, while the substitution position of the methyl group in the benzothiazole ring had no significant effect on the antiviral activity of the compound. For example, the inhibition rate of compound **71** on TMV was 45.1% [104]. The treatment activity of compound **72** against TMV was 39.3%. When the oxazole ring was replaced by a thiazole ring, the antiviral activity of the compound increased. For example, the treatment activity of compound **73** against TMV was 52% [105]. The protective and passivation activities of compound **74** against TMV were 78.3% and 79.5%, and the protective and passivation activities of compound **75** against TMV were 83.3%. The replacement of chlorine atoms with nitro atoms did not significantly change the antiviral activity of the compound [55].

## 5. Herbicidal Activity

Weeds compete with crops for nutrients, sunlight, and water, harming the normal growth and yield of crops. Furthermore, some weeds contain toxins in their seeds or pollen that can harm human health [106,107]. The use of chemical herbicides is the most effective and cost-effective way to manage weeds [108,109]. Currently, 263 species of weeds worldwide have shown resistance to 23 herbicides [110,111]. Therefore, the discovery of new herbicides is an urgent need for weed management [112,113].

**Figure 9:**
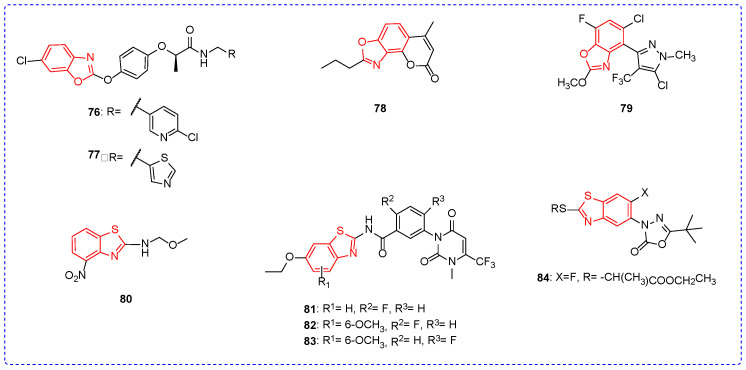
Chemical structures analysis of benzoxazole and benzothiazole herbicidal active compounds **76**–**84**.

Although the herbicidal activities of benzoxazole and benzothiazole derivatives have been less reported, some compounds have shown excellent herbicidal activities. For example, compounds **76** and **77** (Figure 9) both achieved 90% (Table 8) herbicidal activity against the monocotyledon weeds *Digitaria sanguinalis* and *Setaria viridis* at a concentration of 75 g/ha [114]. In addition, compounds **76** and **77** showed good safety on the stems and leaves of rice. At a concentration of 100 μg/L, compound **78** had 93% and 85% herbicidal activities against the roots and stems of *Chenopodium album (C. album)*, respectively. In addition, compound **78** may show herbicidal activity by inhibiting the growth of the taproot and stem of the *C. album* [68]. Under the condition of 37.5 g/hm^2^, compound **79** showed 100% inhibition rate against *Setaria viridis, Ditaria sanguinalis,* and *Abutilon theophrasti*. The introduction of the alkoxy group was beneficial to increase the herbicidal activity of the compound [115]. The inhibition rate of compound **80** to *Amaranthus retroflexus (A. retroflexus)* was 100% at 1400 g/ha, and the introduction of the nitro group improved the herbicidal activity of the compound [116]. Compounds **81**, **82**, and **83** showed 99% herbicidal activities against *A. retroflexus* at a concentration of 10 mg/L, and the introduction of fluorine may have increased the herbicidal activity of the compounds [117]. Under the condition of 37.5 g/hm^2^, the inhibition rate of compound **84** against *Abutilon theophrasti, Cyperus iria, Rumex acetasa,* and *Eclipta prostrate* was greater than 80%, which has the prospect of further development [118].

## 6. Insecticidal Activity

**Figure 10:**
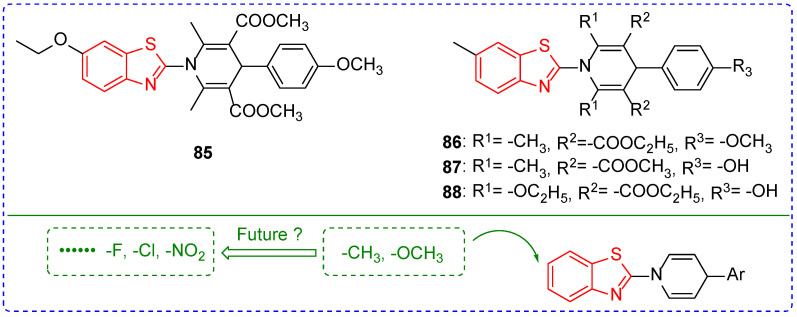
Chemical structures and modified fragments analysis of benzothiazole insecticidal active compounds **85**–**88**.

The wide variety of pests is an important factor in crop yield reduction and some pests are characterized by the outbreak, such as *Pyrausta nubilalis* [119], *Helicoverpa armigera* [120], *Oriental armyworm* [121,122], and *Locust* [123,124,125]. Traditional insecticides have played an irreplaceable role in pest control, and the long-term use of traditional insecticides not only leads to the rapid increase in pest resistance but also pollutes the environment and threatens human health [126,127,128]. The discovery of insecticides has always been a hot topic in pesticide research [129]. However, there are relatively few reports on the insecticidal activity of benzoxazole and benzothiazole, which may be strengthened in the future. The Maximum Likelihood Programmer (MLP) calculation showed that the combination of benzothiazole and pyridine could increase the antifeedant activity of the compounds. For example, LC_50_ of compounds **85**–**88** (Figure 10) against *Spodoptera litura* were 0.38, 0.24, 0.10, and 0.07, respectively [130,131]. The insecticidal activity of compounds **86**, **87**, and **88** was significantly higher than that of compound **85**, which may be due to the different electronegativity of groups introduced at position-6 of benzothiazole. Perhaps this is a hint that we can try to introduce strong electron-absorbing groups such as nitro and trifluoromethyl to benzothiazole in the future to find new insecticides.

**Figure 11:**
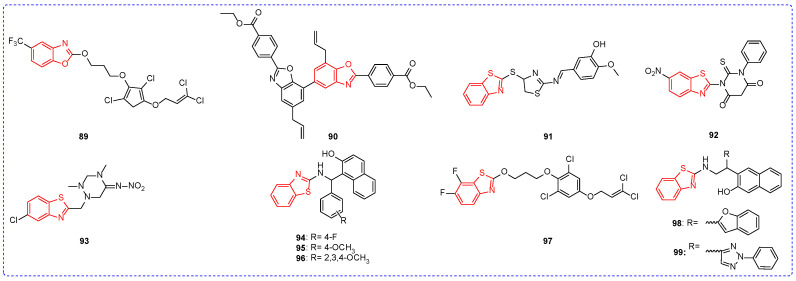
Chemical structures of benzothiazole insecticidal active compounds **89**–**99**.

At a concentration of 1 mg/L, the insecticidal activity of compound **89** (Figure 11) against *Spodoptera exigua* was 100% (Table 9); perhaps the strong electron-absorbing group trifluoromethyl played an important role in the insecticidal activity of compound **89 [132]**. The insecticidal activity of compound **90** against *Mythimna separata Walker* was 62.1%, which was better than that of the lead compound magnolol [133]. Under the concentration of 5 g/L, the mean killing time of compound **91** to cockroaches was 147 min, which was better than that of commercial Parathion (280 min) [134]. The LC_50_ of compound **92** for *Tetranychus urticae* was 0.07 mg/L [135]. The insecticidal activity of compound **93** against *Aphis* was 54% at a concentration of 200 mg/mL [136]. The ED_50_ value of compound **94** for *Achaea janata (A. janata)* was 19.3 μg/cm^2^. The insecticidal activity of the compounds was significantly improved when fluorine atoms on the benzene ring were replaced with methoxide. For example, compounds **95** and **96** had ED_50_ values of 7.0 and 5.2 μg/cm^2^ for *A. janata*, respectively. Meanwhile, the insecticidal activities of compounds **95** and **96** against *Spodoptera litura* were greater than 95% at a concentration of 0.2 μg/insect [137]. The LC_50_ value of compound **97** against *Bollworm* was 4.90 mg/L [138]. The insecticidal activity of compound **98** against the *Diamondback moth* was 88% at a concentration of 1 mg/L. In addition, at high concentrations, compound **99** showed good insecticidal activity by activating the release of calcium ions from the central neurons of insects [139].

## 7. Conclusions

Benzothiazoles and benzoxazoles not only have a bicyclic structure, but also have seven modifiable sites, illustrating the important value of benzothiazoles and benzoxazoles in the discovery of pesticides. It is worthy to carry out more exploration and research based on benzothiazoles or benzoxazoles. In recent years, benzoxazole and benzothiazole derivatives have been increasingly studied as fungicides, antimicrobials, herbicides, antiviral agents, and insecticides. However, the research on the mechanism of action and the discovery of new targets of benzoxazole and benzothiazole derivatives compounds is still weak and needs to be further strengthened in the future, which is a key factor restricting the discovery of new green pesticides. We systematically reviewed the application of benzoxazole and benzothiazole derivatives compounds in the discovery of new agrochemicals, summarized the antibacterial, fungicidal, and antiviral agents, as well as herbicidal and insecticidal activities, of the compounds, and discussed the structural–activity relationship and mechanism of action of the active compounds, aiming to provide new clues and inspiration for the discovery of new pesticides.

## Figures and Tables

**Table 1:** Benzoxazole or benzothiazole antibacterial derivatives with antifungal activity.

Compound	Bacteria	Concentration	Antibacterial Activity	SAR/Physiology and Biochemistry
**1**	*Xoc* *Xac*		47.6 ^a^ 36.8 ^a^	The expression of SDH during oxidative phosphorylation is up-regulated.
**2**	*Xoo*	100 mg/L	52.4%	
**3**	*Rs*	100 mg/L	71.6%	The introduction of the nitro group and trifluoromethyl group plays a key role.
**4**	*Xoo* *Xac* *Rs*	100 mg/L	52.40% 50.97% 36.49%,	The introduction of electron-withdrawing groups enhances the antibacterial activity of the compounds.
**7**	*Xoo*		11.4 ^a^	Cell morphology is altered and biofilm formation is inhibited.
**10**	*Xoo*		20.0 ^a^	The introduction of the fluorine atom plays a key role.
**11**	*Xac*		35.7 ^a^	The cell surface morphology is folded and damaged.
**12**	*Xac*		28.5 ^a^	

^a^ median effective concentration (EC_50_, mg/L).

**Table 2:** Benzoxazole derivatives with antifungal activity.

Compound	Fungus	Concentration	Antifungal Activity	SAR/Molecular Docking
**13**	*Alternaria brassicae*		0.3 ^a^	
**14**	*Botrytis cinerea*	90 mg/L	>88%	
**16**	*Botrytis cinerea*		2.40 ^a^	Compound 16 may show fungicidal activity by binding to the active site of the sec14p target of fungi
**18**	*Botrytis cinerea*		1.69 ^a^	The introduction of electron-absorbing groups is beneficial for antifungal activity.

^a^ median effective concentration (EC_50_, mg/L).

**Table 3:** Benzothiazole derivatives with antifungal activity.

Compound	Fungus	Concentration	Antifungal Activity	SAR
**19**	*Botrytis cinerea*		1.4 ^a^	The addition of methylene between benzothiazole and aryl increased the fungicidal activity of the compound
**20**	*Rhizoctonia solani*, *Botrytis cinereal*, *Dothiorella gregaria*, *Colletotrichum gossypii*	50 mg/L	92% 97% 89% 78%	
**22**	*Sphaerotheca fuliginea*, *Pseudoperoniospora cubensis*		6.17 ^b^ 46.32 ^b^	
**23**	*Sphaerotheca fuliginea*, *Pseudoperoniospora cubensis*	50 mg/L	67% 67%	The introduction of large steric groups reduces the fungicidal activity of the compound.
**25** **27** **29**	*Phytophthora infestans*	100 ppm	58% 53% 58%	The fungicidal activity improves when chlorine atoms on position-2 of the benzene ring are replaced by position-4 fluorine atoms.

^a^ half maximal inhibitory concentration (IC_50_, μmol/L). ^b^ concentration for 90% of maximal effect (EC_90_, mg/L).

**Table 4:** Benzothiazole derivatives with antifungal activity.

Compound	Fungus	Concentration	Antifungal Activity	SAR
**30**	*Botrytis cinereal*	1000 mg/L	88.9%	The introduction of the nitrophenyl group increases antifungal activity.
**31**	*Botrytis cinereal*, *Gibberella zeae*	50 mg/L	80% 75%	The introduction of permethric acid had no significant contribution to the fungicidal activity of the compound
**32**	*Ustilago tritici*, *Puccinia striiformis*, *Puccinia triticina*, *Blumeria graminis*, *Dickeya oryzae* *Ustilag ohordeiare*		<0.8 ^a^	
**35**	*Rhizoctonia solani*, *Botrytis cinereal*, *Gibberella zeae*, *Helminthosporium maydis*, *Sclerotinia sclerotiorum*	50 mg/L	93.8%, 94.1%, 93.4%, 94.6%, 94.5%	The introduction of oxazoles plays a key role
**36**	*Fusarium oxysporum*		12.5 ^b^	

^a^ median effective concentration (EC_50_, mmol/L). ^b^ minimum inhibitory concentration (MIC, mg/mL).

**Table 5:** Benzothiazole derivatives with antifungal activity.

Compound	Fungus	Concentration	Antifungal Activity
**37**	*Alternaria alternate*, *Aspergillus niger*	100 mg/L	38% 39%
**38**	*Gibberella zeae*	250 mg/L	53.5%
**39**	*Sclerotinia sclerotiorum*	100 mg/L	87.5%
**41**	*Rhizoctonia solani*	50 mg/L	70.43%
**42**	*Fusarium oxysporum*	50 mg/L	60.53%
**43**	*Aspergillus oryzae*	10 mg/L	0.81 ^a^
**46**	*Rape sclerotinia rot*	50 mg/L	81.61%
**47**	*Rhizoctonia solani*		0.96 ^b^

^a^ The inhibitory zone diameter(mm). ^b^ median effective concentration (EC_50_, µM).

**Table 6:** Benzothiazole derivatives with antiviral activity.

Compound	Virus	Concentration	Antiviral Activity	SAR
**50**	TMV	500 mg/L	54.41% ^a^	The electron-donating group in the benzothiazole ring may play a key role
**52**	TMV	500 mg/L	55.96% ^b^	The introduction of the chlorine atom plays a key role
**54**	TMV	500 mg/L	28.2%	
**57**	TMV	500 mg/L	48.1% ^a^	
**58**	TMV	500 mg/L	48.2% ^a^	
**61**	TMV	500 mg/L	74.3% ^a^ 78.7% ^b^ 94.3% ^c^	The hydrazone’s structure can affect the compounds’ antiviral activity.

^a^ treatment activity, ^b^ protective activity, ^c^ passivation activity.

**Table 7:** Benzothiazole derivatives with antiviral activity.

Compound	Virus	Concentration	Antiviral Activity	SAR
**62**	TMV	500 mg/L	52.9% ^a^	Straight-chain alkane is beneficial to the antiviral activity of the compound
**67**	TMV	500 mg/L	44.0%	The introduction of the benzene ring is beneficial to the improvement of the anti-TMV activity of the compound
**68**	CMV	50 mg/L	46.3%	The growth of the alkyl chain had little effect on the antiviral activity of the compound
**71**	TMV	500 mg/L	45.1%	
**73**	TMV	500 mg/L	52%	The introduction of the thiazole ring is beneficial to the antiviral activity of the compound
**75**	TMV	500 mg/L	83.3% ^b,c^	

^a^ treatment activity, ^b^ protective activity, ^c^ passivation activity.

**Table 8:** Benzoxazole and benzothiazole derivatives with herbicidal activity.

Compound	Weeds	Concentration	Herbicidal Activity	SAR/Physiology and Biochemistry
**76**	*Digitaria sanguinalis*, *Setaria viridis*	75 g/ha	90%	The compound shows good safety on the stems and leaves of rice
**78**	the roots of *Chenopodium album*, the stems of *Chenopodium album*	100 μg/L	93% 85%	The compound inhibits the growth of the taproot and stem of the *Chenopodium album*
**79**	*Setaria viridis*, *Ditaria sanguinalis*, *Amaranthus retroflexus*	37.5 g/hm^2^	100%	The introduction of the alkoxy group was beneficial to increase the herbicidal activity.
**81**	*Amaranthus retroflexus*	10 mg/L	99%	The introduction of fluorine may have increased the herbicidal activity of the compounds
**84**	*Abutilon theophrasti*, *Cyperus iria*, *Rumex acetasa*, *Eclipta prostrate*	37.5 g/hm^2^	>80%	

**Table 9:** Benzoxazole and benzothiazole derivatives with insecticidal activity.

Compound	Pests	Concentration	Insecticidal Activity	SAR
**88**	*Spodoptera litura*		0.07 ^a^	The introduction of the ethoxy group may play a key role
**89**	*Spodoptera exigua*	1 mg/L	100%	The strong electron-absorbing group may play a key role
**90**	*Mythimna separata Walker*	1 mg/L	62.1%	
**91**	cockroaches	5 g/L	147 ^b^	
**92**	*Tetranychus urticae*		0.07 ^c^	
**93**	*Aphis*	200 mg/mL	54%	
**96**	*Achaea janata*		5.2 ^d^	Fluorine atom on the benzene ring improves the insecticidal activity of the compound
**97**	*Bollworm*		4.90 ^c^	
**98**	*Diamondback moth*	1 mg/L	88%	

^a^ the calculation of LC_50_/LD_50_ using the Maximum Likelihood Programmer (MLP). ^b^ the mean killing time (min). ^c^ lethal concentration 50 (LC_50,_ mg/L). ^d^ a median effective concentration (EC_50_, μg/cm^2^).

## Data Availability

The data that support the findings of this study are available from the corresponding author upon reasonable request.

## Abbreviations

SAR	Structural–activity relationship
*Xoc*	*Xanthomonas oryzae* pv. *oryzicola*
*Xac*	*Xanthomonas citri* subsp. *Citri*
*Xoo*	*Xanthomonas oryzae* pv. *oryzae*
SDH	Succinate dehydrogenase
*Rs*	*Ralstonia solanacearum*
*B. cinerea*	*Botrytis cinereal*
*R. solani*	*Rhizoctonia solani*
*D. gregaria*	*Dothiorella gregaria*
*C. gossypii*	*Colletotrichum gossypii*
*S. fuligine* *a*	*Sphaerotheca fuligine* *a*
*P. cubensis*	*Pseudoperoniospora cubensis*
*P. infestans*	*Phytophthora infestans*
*G. zeae*	*Gibberella zeae*
*S. sclerotiorum*	*Sclerotonia sclerotiorum*
*F. oxysporum*	*Fusarium oxysporum*
*A. oryzae*	*Aspergillus oryzae*
TMV	tobacco mosaic virus
CMV	Cucumber mosaic virus
*C. album*	*Chenopodium album*
*A. retroflexus*	*Amaranthus retroflexus*
*A. janata*	*Achaea janata*
MLP	Maximum Likelihood Programmer

## References

[1] Strange R.N., Scott P.R. (2005). Plant disease: A threat to global food security. Annu. Rev. Phytopathol..

[2] Chen J.X., Wang Y., Luo X., Chen Y.F. (2022). Recent research progress and outlook in agricultural chemical discovery based on quinazoline scaffold. Pestic. Biochem. Physiol..

[3] Möller M., Stukenbrock E.H. (2017). Evolution and genome architecture in fungal plant pathogens. Nat. Rev. Microbiol..

[4] Evidente A., Cimmino A., Masi M. (2019). Phytotoxins produced by pathogenic fungi of agrarian plants. Phytochem. Rev..

[5] Chen Y.F., Luo X., Wang Y., Xing Z.F., Peng J., Chen J.X. (2023). Design, synthesis and antibacterial activity of 1,3,4-oxadiazole sufones containing sulfonamide structure. Chin. J. Org. Chem..

[6] Leonard S., Hommais F., Nasser W., Reverchon S. (2016). Plant–phytopathogen interactions: Bacterial responses to environmental and plant stimuli. Environ. Microbiol..

[7] Wei C.Q., Huang J.J., Wang Y., Chen Y.F., Luo X., Wang S.B., Wu Z.X., Xing Z.F., Chen J.X. (2021). Discovery of novel dihydrolipoamide S-Succinyltransferase inhibitors based on fragment virtual screening. Int. J. Mol. Sci..

[8] Chen J.X., Luo X., Chen Y.F., Wang Y., Peng J., Xing. Z.F. (2022). Recent research progress: Discovery of anti-plant virus agents based on natural scaffold. Front. Chem..

[9] Hernan G.R. (2018). Susceptibility genes to plant viruses. Viruses.

[10] Gougherty V.A., Jonathan D.T. (2021). Towards a phylogenetic ecology of plant pests and pathogens. Phil. Trans. R. Soc..

[11] Nelson D.C. (2021). The mechanism of host-induced germination in root parasitic plants. Plant Physiol..

[12] Morin L. (2020). Progress in biological control of weeds with plant pathogens. Annu. Rev. Phytopathol..

[13] Chen J.X., Song B.A. (2021). Natural nematicidal active compounds: Recent research progressand outlook. J. Integr. Agric..

[14] Chen J.X., Wei C.Q., Wu S.K., Luo Y.Q., Wu R., Hu D.Y., Song B.A. (2020). Novel 1,3,4-oxadiazole thioether derivatives containing flexible-chain moiety: Design, synthesis, nematocidal activities, and pesticide-likeness analysis. Bioorg. Med. Chem. Lett..

[15] Chen J.X., Chen Y.Z., Gan X.H., Song B.J., Hu D.Y., Song B.A. (2018). Synthesis, nematicidal evaluation, and 3D-QSAR analysis of novel 1,3,4-oxadiazole-cinnamic acid hybrids. J. Agric. Food Chem..

[16] Kim S., Yoon K.A., Cho S., Lee J., Lim Y., Lee S.H. (2022). Molecular and kinetic properties of three acetylcholinesterases in the Varroa mite, Varroa destructor. Pestic. Biochem. Physiol..

[17] Goeb J., Lupi F. (2021). Showing pesticides’ true colors: The effects of a farmer-to-farmer training program on pesticide knowledge. J. Environ. Manag..

[18] Wei C.Q., Huang J.J., Luo Y., Wang S.B., Wu S.K., Xing Z.F., Chen J.X. (2021). Novel amide derivatives containing an imidazo[1,2-a]pyridine moiety: Design, synthesis as potential nematicidal and antibacterial agents. Pestic. Biochem. Physiol..

[19] Nagoshi R.N., Meagher R.L. (2022). The Spodoptera frugiperda host strains: What they are and why they matter for understanding and controlling this global agricultural pest. J. Econ. Entomol..

[20] Zhang L., Lecoq M., Latchininsky A., Hunter D. (2019). Locust and Grasshopper Management. Annu. Rev. Entomol..

[21] Wang J., Chen L., Lin D., Zhang J., Zhao J., Xiao D., Wang R., Wang R., Gao S. (2019). Molecular cloning, characterization and functional analysis of GluCl from the oriental armyworm, *Mythimna separata* Walker. Pestic. Biochem. Physiol..

[22] Torto B., Cortada L., Murungi L.K., Haukeland S., Coyne D.L. (2018). Management of cyst and root knot nematodes: A chemical ecology perspective. J. Agric. Food Chem..

[23] Meftaul I.M., Venkateswarlu K., Dharmarajan R., Annamalai P., Asaduzzaman M., Parven A., Megharaj M. (2020). Controversies over human health and ecological impacts of glyphosate: Is it to be banned in modern agriculture?. Environ. Pollut..

[24] Pu J., Wang Z., Chung H. (2020). Climate change and the genetics of insecticide resistance. Pest Manag. Sci..

[25] Chen J.X., Luo Y.Q., Wei C.Q., Wu S.K., Wu R., Wang S.B., Hu D.Y., Song B.A. (2020). Novel sulfone derivatives containing a 1,3,4-oxadiazole moiety: Design and synthesis based on the 3D-QSAR model as potential antibacterial agent. Pest Manag. Sci..

[26] Skrzypek A., Karpińska M., Juszczak M., Grabarska A., Wietrzyk J., Krajewska-Kułak E., Studziński M., Paszko T., Matysiak J. (2022). Cholinesterases inhibition, anticancer and antioxidant activity of novel benzoxazole and naphthoxazole analogs. Molecules.

[27] Zhang Y., Plattner J.J., Easom E.E., Waterson D., Ge M., Li Z., Li L., Jian Y. (2011). An efficient synthesis for a new class antimalarial agent, 7-(2-carboxyethyl)-1,3-dihydro-1-hydroxy-2,1-benzoxaborole. Tetrahedron Lett..

[28] Zhou M.Z., Wang W.W., Wang Z.K., Wang Y.L., Zhu Y.F., Lin Z.Q., Tian S.Q., Huang Y., Hu Q.H., Li H. (2022). Discovery and computational studies of 2-phenyl-benzoxazole acetamide derivatives as promising P2Y14R antagonists with anti-gout potential. Eur. J. Med. Chem..

[29] Bhat M., Belagali S.L. (2020). Structural activity relationship and importance of benzothiazole derivatives in medicinal chemistry: A comprehensive review. Mini Rev. Org. Chem..

[30] Lányi K., Laczay P., Lehel J. (2016). Effects of some naturally occurring substances on the photodegradation of herbicide methabenzthiazuron. J. Environ. Chem. Eng..

[31] Kyung K.S., Ahn K.C., Kwon J.W., Lee Y.P., Lee E.Y., Kim Y.J., Führ F., Lee J.K. (2015). Long-term fate of the herbicide mefenacet in a rice-grown lysimeter over a period of 6 consecutive years. J. Appl. Biol. Chem..

[32] Jin L.H., Song B.A., Zhang G.P., Xu R.Q., Zhang S.M., Gao X.W., Hu D.Y., Yang S. (2006). Synthesis, x-ray crystallographic analysis, and antitumor activity of *N*-(benzothiazole-2-yl)-1-(fluorophenyl)-*O,O*-dialkyl-alpha-aminophosphonates. Bioorg. Med. Chem. Lett..

[33] Sondhi S.M., Singh N., Kumar A. (2006). Synthesis, anti-inflammatory, analgesic and kinase (CDK-1, CDK-5 and GSK-3) inhibition activity evaluation of benzimidazole/benzoxazole derivatives and some Schiff’s bases. Bioorg. Med. Chem. Lett..

[34] Sigmundova I., Zahradnik P., Magdolen P., Bujdakova H. (2008). Synthesis and study of new antimicrobial benzothiazoles substituted on heterocyclic ring. Arkivoc.

[35] Mortimer C.G., Wells G., Crochard J.P., Stone E.L., Bradshaw T.D., Stevens M.F.G., Westwell A.D. (2006). Antitumor Benzothiazoles. 26. 2-(3,4-Dimethoxyphenyl)-5-fluorobenzothiazole (GW 610, NSC 721648), a Simple Fluorinated 2-Arylbenzothiazole, Shows Potent and Selective Inhibitory Activity against Lung, Colon, and Breast Cancer Cell Lines. J. Med. Chem..

[36] Barik S.R., Ganguly P., Patra S., Dutta S.K., Goon A., Bhattacharyya A. (2018). Persistence behavior of metamifop and its metabolite in rice ecosystem. Chemosphere.

[37] Zhu W., He Y., Li L. (2011). Effects of 10%metamifop wp on gramineous weeds control in direct seeding paddy field. Weed Sci..

[38] Smith A.E. (1985). Persistence and transformation of the herbicides [14C] fenoxaprop-ethyl and [14C] fenthiaprop-ethyl in two prairie soils under laboratory and field conditions. J. Agric. Food Chem..

[39] Cai X., Chen J., Wang X., Gao H., Xiang B., Dong L. (2022). Mefenacet resistance in multiple herbicide-resistant *Echinochloa crus-galli* L. populations. Pestic. Biochem. Phys..

[40] Liu Y., Yang J., Chai B., Wu Q., Liu C. (2011). A Novel fungicide benthiavalicarb-isopropyl. Agrochemicals.

[41] Bi Q., Ma Z. (2016). Sensitivity, resistance stability, and cross-resistance of *Plasmopara viticola* to four different fungicides. Crop Prot..

[42] Wang D., Xie X., Gao D., Chen K., Chen Z., Jin L., Li X., Song B. (2019). Dufulin intervenes the viroplasmic proteins as the mechanism of action against southern rice black-streaked dwarf virus. J. Agric. Food Chem..

[43] Chen Z., Zeng M., Song B., Hou C., Hu D., Li X., Wang Z., Fan H., Bi L., Liu J. (2012). Dufulin activates HrBP1 to produce antiviral responses in tobacco. PLoS ONE.

[44] Li X.Y., Cheng Z., Yang S., Song B.A. (2010). Research progress of structure biology of tobacco mosaic virus coat protein as molecular targets. Chin. J. Pestic. Sci..

[45] Ma G., Zhang Y., Li X. (2022). Dufulin enhances salt resistance of rice. Pestic. Biochem. Physiol..

[46] Wang X.Y., Chen S.H., Zhang J., Gao Y.X., Zhang L.X. (2022). Research progress of insecticide oxazosulfyl and its analogues. Contemp. Chem. Ind..

[47] Suzuki T., Yamato S. (2021). Oxazosulfyl, a novel sulfyl insecticide, binds to and stabilizes the voltage-gated sodium channels in the slow-inactivated state. J. Agr. Food Chem..

[48] Chen J.X., Yi C.F., Wang S.B., Wu S.K., Li S.Y., Hu D.Y., Song B.A. (2019). Novel amide derivatives containing 1,3,4-thiadiazole moiety: Design, synthesis, nematocidal and antibacterial activities. Bioorg. Med. Chem. Lett..

[49] Wang S., Chen J., Shi J., Wang Z., Hu D., Song B. (2021). Novel cinnamic acid derivatives containing the 1,3,4-oxadiazole moiety: Design, synthesis, antibacterial activities, and mechanisms. J. Agric. Food Chem..

[50] Mou H., Shi J., Chen J., Hu D. (2021). Synthesis, antibacterial activity and mechanism of new butenolides derivatives containing an amide moiety. Pestic. Biochem. Physiol..

[51] Sundin G.W., Wang N. (2018). Antibiotic resistance in plant-pathogenic bacteria. Annu. Rev. Phytopathol..

[52] Ji Z., Wang C., Zhao K. (2018). Rice Routes of Countering *Xanthomonas oryzae*. Int. J. Mol. Sci..

[53] Wu S., Shi T., Chen J., Hu D., Zang L., Song B. (2021). Synthesis, antibacterial activity, and mechanisms of novel 6 sulfonyl-1,2,4-triazolo[3,4 b][1,3,4]thiadiazole derivatives. J. Agric. Food Chem..

[54] Liu T., Shi J., Liu D., Zhang D., Song B., Hu D. (2022). Discovery of novel benzo[4,5]thiazolo(oxazolo)[3,2-a]pyrimidinone mesoionic derivatives as potential antibacterial agents and mechanism research. J. Agric. Food Chem..

[55] Tang X., Wang Z., Zhong X., Wang X., Chen L., He M., Xue W. (2019). Synthesis and biological activities of benzothiazole derivativesbearing a 1,3,4-thiadiazole moiety. Phosphorus Sulfur Silicon Relat. Elem..

[56] Wang Y., Li P., Jiang S., Chen Y., Su S., He J., Chen M., Zhang J., Xu W., He M. (2019). Synthesis and antibacterial evaluation of novel chalcone derivatives containing a benzothiazole scaffold. Monatsh. Chem..

[57] Zhang J., Wei C., Li S., Hu D., Song B. (2020). Discovery of novel bis-sulfoxide derivatives bearing acylhydrazone and benzothiazole moieties as potential antibacterial agents. Pestic. Biochem. Physiol..

[58] Wang Y., Zhou R., Sun N., Sun M., He M., Wu Y., Xue W. (2021). Synthesis and antibacterial activity of novel 1,4-pentadien-3-one derivatives bearing a benzothiazole moiety. J. Heterocycl. Chem..

[59] Kang Z.S. (2010). Current status and development strategy for research on plant fungal diseases in China. Plant Protect..

[60] El-Baky N.A., Amara A.A.A.F. (2021). Recent approaches towards control of fungal diseases in plants: An updated review. J. Fungi.

[61] Luo X., Chen Y.F., Wang Y., Xing Z., Peng J., Chen J.X. (2023). Design, synthesis and antifungal activity of novel amide derivatives containing a pyrrolidine moiety as potential succinate dehydrogenase inhibitors. Mol. Divers..

[62] Ons L., Bylemans D., Thevissen K., Cammue B.P. (2020). Combining biocontrol agents with chemical fungicides for integrated plant fungal disease control. Microorganisms.

[63] Wei Y., Li S., Hao S. (2018). New angular oxazole-fused coumarin derivatives: Synthesis and biological activities. Nat. Prod. Res..

[64] Xu H., Wei Y., Hao S. (2022). 4-Methylumbelliferone fused oxazole thioether derivatives: Synthesis, characterization and antifungal activities. Nat. Prod. Res..

[65] Fan L., Luo Z., Yang C., Guo B., Miao J., Chen Y., Tang L., Li Y. (2022). Design and synthesis of small molecular 2-aminobenzoxazoles as potential antifungal agents against phytopathogenic fungi. Mol. Divers..

[66] Ballari M.S., Cano N.H., Lopez A.G., Wunderlin D.A., Feresin G., Santiago A.N. (2017). Green Synthesis of potential antifungal agents: 2-benzyl substituted thiobenzoazoles. J. Agric. Food Chem..

[67] Huang W., Yang G. (2006). Microwave-assisted, one-pot syntheses and fungicidal activity of polyfluorinated 2-benzylthiobenzothiazoles. Bioorg. Med. Chem..

[68] Huang W., Zhao P., Liu C., Chen Q., Liu Z., Yang G. (2007). Design, synthesis, and fungicidal activities of new strobilurin derivatives. J. Agric. Food Chem..

[69] Zhao P., Wang F., Huang W., Chen Q., Liu Z. (2010). Synthesis and Fungicidal Activities of Novel Thioethers Containing Benzothiazole Moiety. Chin. J. Org. Chem..

[70] Singh R.P., Singh D.V., Singh C.R., Tripathi S.P., Singh S. (2012). Synthesis and antifungal activity of 2-azetidinones, 4-thiazolidinones and 5-imidazolidinones incorporating benzthiazole moiety. Chem. Pharm. Res..

[71] Wang Y., Song H.Y., Wang S., Cai Q.F., Zhang Y., Zou Y., Liu X., Chen J.X. (2023). Discovery of quinazoline compound as a novel nematicidal scaffold. Pestic. Biochem. Physiol..

[72] Hou X., Wang M., Jing S., Niu S. (2006). Synthesis and induced resistance activities of methyl 2-amidobenzothiazole-7-carboxylates. Chin. J. Pestic. Sci..

[73] Zhao J., Zhou Y., Xu X., Lie Z., Zhu G., Jing C. (2010). Synthesis and biological activities of *N*-(substituted-thiazole-2-yl)-chrysanthemumamide. Chin. J. Org. Chem..

[74] Sidhu A., Kukreja S. (2019). Synthesis of novel fluorinated benzothiazol-2-yl-1,2,4-triazoles: Molecular docking, Antifungal evaluation and in silico evaluation for SAR. Arab. J. Chem..

[75] Wang X., Gao S., Yang J., Gao Y., Wang L., Tang X. (2015). Synthesis and antifungal activity evaluation of new heterocycle containing amide derivatives. Nat. Prod. Res..

[76] Gao Y., Yang J., Gao S., Wang X., Tang X. (2016). Synthesis of *N*-(benzod]thiazol-2-yl) furan-2-carboxamide and Research on Its Antifungal Activity. J. Xihua Univ..

[77] Bondock S., Fadaly W., Metwally M.A. (2010). Synthesis and antimicrobial activity of some new thiazole, thiophene and pyrazole derivatives containing benzothiazole moiety. Eur. J. Med. Chem..

[78] Sareen V., Khatri V., Garg U., Jain P., Sharma K. (2007). Synthesis of Some New 2-(4-Nitrobenzothiazol-2′-ylamino)-4-(2-chloro-4-trifluoromethylanilino)-6-(substituted Thioureido)-1,3,5-triazines as Antifungal Agents. Phosphorus Sulfur Silicon Relat. Elem..

[79] Lv X., Zhang X., Tang W. (2010). Synthesis and antibacterial activity of cinnamic acyl thiourea derivatives. Appl. Chem..

[80] Weng J., Huang H., Ta C., Liu X., Chu W., Chen J. (2012). Synthesis and antifungal activity of novel substituted-3-aryl-1,2,4-triazolo[3,4-b]benzothiazoles. Chin. J. Org. Chem..

[81] Weng J., Tan C., Liu X. (2012). Synthesis and fungicidal activity of hydrazones containing 4-methylbenzod]thiazole moiety. J. Pestic. Sci..

[82] Xiao H., Wu F., Shi L., Chen Z.W., Su S.H., Tang C.H., Wang H.T., Li Z.N., Li M.C., Shi Q.C. (2014). Cinchona alkaloid derivative-catalyzed enantioselective synthesis via a Mannich-type reaction and antifungal activity of *β*-amino esters bearing benzoheterocycle moieties. Molecules.

[83] Zeng Z., Huang Q., Wei Y., Huang Q., Wang Q. (2017). Synthesis, crystal structure and antibacterial activity of thiourea derivatives with benzothiazole-ring. Chem. Reagents.

[84] Huo X., Li W., Zhang B., Chen X., Zhou Y., Zhang J., Han X., Dai B. (2018). Synthesis and fungicidal evaluation of novel β-carboline benzimidazole and β-carboline-benzothiazole hybrids. Chin. J. Org. Chem..

[85] Upadhyay R.K., Saini K.K., Deswal N., Singh T., Tripathi K.P., Kaushik P., Shakil N.A., Bharti A.C., Kumar R. (2022). Synthesis of benzothiazole-appended bis-triazole-based structural isomers with promising antifungal activity against *Rhizoctonia solani*. RSC Adv..

[86] Jones R.A.C. (2021). Global plant virus disease pandemics and epidemics. Plants.

[87] Liu Y., Chen J., Xie D., Song B., Hu D. (2021). First report on anti-TSWV activities of quinazolinone derivatives containing a dithioacetal moiety. J. Agric. Food Chem..

[88] He F., Shi J., Wang Y., Wang S., Chen J., Gan X., Song B., Hu D. (2019). Synthesis, Antiviral activity, and mechanisms of purine nucleoside derivatives containing a sulfonamide moiety. J. Agric. Food Chem..

[89] Tatineni S., Hein G.L. (2023). Plant viruses of agricultural importance: Current and future perspectives of virus disease management strategies. Phytopathology.

[90] Calil I.P., Fontes E.P. (2017). Plant immunity against viruses: Antiviral immune receptors in focus. Ann. Bot..

[91] Xiao Q.Z., Deng B., Zou H.L., Teng Q.J., Zhou Z.C. (2021). Study progress in biological prevention of plant virus. South China Agric..

[92] Yu W.X., Shen J.Z. (2021). An overview on plant viral diseases and antiviral agents. World Pestic..

[93] Zhang J., He F., Chen J., Wang Y., Yang Y., Hu D., Song B. (2021). Purine nucleoside derivatives containing a sulfa ethylamine moiety: Design, synthesis, antiviral activity, and mechanism. J. Agric. Food Chem..

[94] Jiang D., Chen J., Zan N., Li C., Hu D., Song B. (2021). Discovery of novel chromone derivatives containing a sulfonamide moiety as anti-ToCV agents through the tomato chlorosis virus coat protein-oriented screening method. Bioorg. Med. Chem. Lett..

[95] Han X., Zheng P.C., Tang C.P., Xie Y., Wu F., Song J., Zhao J., Li Z.N., Li M.C. (2013). Synthesis and anti-TMV activity of dialkyl/dibenzyl 2-((6-substituted-benzod]thiazol-2-ylamino)(benzofuran-2-yl)methyl) malonates. Molecules.

[96] Xiao H., Li P., Hu D., Song B.A. (2014). Synthesis and anti-TMV activity of novel *β*-amino acid ester derivatives containing quinazoline and benzothiazole moieties. Bioorg. Med. Chem. Lett..

[97] Yao Y.Y., Zhang X., Chen S.X., Xing M.M., Shu H., Tang B.C., Xue W. (2016). Synthesis of benzothiazol-*β*-amino acid esters derivatives applied to research of anti-TMV. Molecules.

[98] Zhang P., Tang C., Chen Z., Wang B., Wang X., Jin L., Yang S., Hu D. (2014). Design, synthesis, and antiviral activity of α-aminophosphonates bearing a benzothiophene moiety. Phosphorus Sulfur Silicon Relat. Elem..

[99] Xie D., Zhang A., Liu D., Yin L., Wan J., Zeng S., Hu D. (2017). Synthesis and antiviral activity of novel a-aminophosphonates containing 6-fluorobenzothiazole moiety. Phosphorus Sulfur Silicon Relat. Elem..

[100] Tian J., Ji R., Wang H., Li S., Zhang G. (2022). Discovery of novel α-aminophosphonates with hydrazone as potential antiviral agents combined with active fragment and molecular docking. Adv. Novel Nat. Prod. Pestic..

[101] Liu L., Song B., Bhadury P.S., Hu D., Yang S. (2012). Synthesis and bioactivities of α-aminophosphonate derivatives containing benzothiazole and thiourea moieties. Phosphorus Sulfur Silicon Relat. Elem..

[102] Yang S., Song B., Liu X., Pang L., Jin L., Wang H., Hu D., Liu G. (2005). Synthesis and anti-tmv activity of 2-(benzod]thiazol-2-ylthio)-1-(2,3,4-trimethoxyphenyl) ethanoxime ester and ether derivativies. Chin. J. Org. Chem..

[103] Ouyang G., Song B., Hu D.Y. (2005). A novel synthesis of (*E*)-3-methylthio-3-substituted arylamino-2-cyanoacrylates under microwave irradiation. Molecules.

[104] Xue W., Li H., Fan H., Xiong Z., He Y., Qi H. (2012). Synthesis and biological activity of anthranilic diamides compounds containing fluorine and benzothiazole unites. J. Univ. Jinan Sci. Tech..

[105] Wang Z.B., Zhu X.S., Liu M., Zhang X., Xue W. (2014). Synthesis and biological activity of oxime ethers of curcumin derivatives. Chin. J. Org. Chem..

[106] Sun J.Q., Ren X.L., Hu H.Y., Jiang W.L., Ma Y.J., Wang D., Song X.P., Ma Y., Ma X.Y. (2019). The factors influencing weed community succession in the crop field. J. Weed Sci..

[107] Zheng Z., Ruhai L., Can Z., Sheng Q. (2021). Reduction in weed infestation through integrated depletion of the weed seed bank in a rice-wheat cropping system. Agron. Sustain. Dev..

[108] Jabusch T., Tjeerdema R. (2008). Chemistry and fate of triazolopyrimidine sulfonamide herbicides. Rev. Environ. Contam. Toxicol..

[109] Chipman D., Duggleby R., Tittmann K. (2005). Mechanisms of acetohydroxyacid synthases. Curr. Opin. Chem. Biol..

[110] Zhou J., Liu K., Xin F., Ma J., Xu N., Zhang W., Fang Y., Jiang M., Dong W. (2018). Recent insights into the microbial catabolism of aryloxyphenoxy-propionate herbicides: Microbial resources, metabolic pathways and catabolic enzymes. World J. Microb. Biot..

[111] Zhang X., Huang Q., Zhao Z., Xu X., Li S., Yin H., Li L., Zhang J., Wang R. (2019). An eco-and user-friendly herbicide. J. Agric. Food Chem..

[112] Neve P., Vila-Aiub M., Roux F. (2009). Evolutionary-thinking in agricultural weed management. New Phytol..

[113] Beckie H.J., Ashworth M.B., Flower K.C. (2019). Herbicide resistance management: Recent developments and trends. Plants.

[114] Liu Q., Hu A.X., Wang X.G., Lei M.X., OU X.M., Ren Y.G., Haung L., Liu A.P. (2014). Synthesis and herbicidal activity of *N*-arylmethyl-2-(4-arylxoyphenoxy) propionamide. Chem. J. Chin. Univ..

[115] Ji Z., Zhou F., Wei S. (2015). Synthesis and herbicidal activities of benzothiazole *N*,*O*-acetals. Bioorg. Med. Chem. Lett..

[116] Che J.Y., Meng X.S., Xu X.Y., Jiang S., Gu Y.C., Shi D.Q. (2016). Synthesis and herbicidal evaluation of novel uracil derivatives containing benzothiazole-2-yl moiety. J. Heterocycl. Chem..

[117] Zhang Y., Chen Y., Xun X., Chen S., Liu Y., Wang Q. (2022). Design, synthesis, acaricidal activities, and structure–activity relationship studies of oxazolines containing ether moieties. J. Agric. Food Chem..

[118] Jiang L.L., Ying T., Zhu X.L., Wang Z.F., Yang Z., Qiong C., Zhen X., Yang G.F. (2010). Design, synthesis, and 3D-QSAR analysis of novel 1,3,4-Oxadiazol-2(3*H*)-ones as protoporphyrinogen oxidase inhibitors. J. Agric. Food Chem..

[119] Rios D.A., Specht A., Roque Specht V.F., Sosa Gómez D.R., Fochezato J., Malaquias J.V., Gonçalves G.L., Moreira G.R. (2022). *Helicoverpa armigera* and *Helicoverpa zea* hybridization: Constraints, heterosis, and implications for pest management. Pest Manag. Sci..

[120] Zhou J., Meng L., Li B. (2019). Non-reproductive effects of two parasitoid species on the oriental armyworm *Mythimna separate* on wheat and maize plants. BioControl.

[121] Sun R., Li Y., Maoyun L., Xiong L., Wang Q. (2010). Synthesis, larvicidal activity, and sar studies of new benzoylphenylureas containing oxime ether and oxime ester group. Bioorg. Med. Chem. Lett..

[122] Shi D., Liang P., Zhang L., Lv H., Gao X., You H., Li J., Ma K. (2022). Susceptibility baseline of Aphis gossypii Glover (Hemiptera: *Aphididae*) to the novel insecticide afidopyropen in China. Crop Prot..

[123] Chen Y.P., Wang Z.D., Fu H.T., Liao L.P., Chen Y., Liu X.F. (2023). Analytical methods and research progress of neonicotinoid insecticides. Fine Chem. Intermed..

[124] Yi C.F., Chen J.X., Wei C.Q., Wu S.K., Wang S.B., Hu D.Y., Song B.A. (2020). α-Haloacetophenone and analogues as potential antibacterial agents and nematicides. Bioorg. Med. Chem. Lett..

[125] Sword G., Lecoq M., Simpson S. (2010). Phase polyphenism and preventative locust management. J. Insect. Physiol..

[126] Chen J., Li Q.X., Song B. (2020). Chemical nematicides: Recent research progress and outlook. J. Agric. Food Chem..

[127] Alexzandrino D.A.M., Almeida C., Marisa R., Mucha A.P., Carvalho M.F. (2022). Revisiting pesticide pollution: The case of fluorinated pesticides. Environ. Pollut..

[128] Lv J., Guo L., Gu Y., Xu Y., Xue Q., Yang X., Wang Q., Meng X., Xu D. (2022). National temporal trend for organophosphate pesticide DDT exposure and associations with chronic kidney disease using age-adapted eGFR model. Environ. Int..

[129] Tian P.Y., Liu D.Y., Liu Z.J., Shi J., He W.J., Qi P.Y., Chen J.X., Song B.A. (2019). Design, synthesis, and insecticidal activity evaluation of novel 4-(*N*,*N*-diarylmethylamines)furan-2(5*H*)-one derivatives as potential acetylcholine receptor insecticides. Pest Manag. Sci..

[130] Mithlesh P.K.P.H. (2010). ChemInform Abstract: Microwave synthesis of new biologically Important 1,4-Dihydropyridines containing benzothiazole moiety. Collect. Czech. Chem..

[131] Pareek P., Kant R., Shukla S., Ojha K. (2010). Rapid synthesis and biological evaluation of 1, 4-dihydropyridine derivatives containing a benzothiazolyl moiety. Open Chem..

[132] Guan A., Qin Y., Wang J., Li B. (2013). Synthesis and insecticidal activity of novel dihalopropene derivatives containing benzoxazole moiety: A structure–activity relationship study. J. Fluor. Chem..

[133] Yang C., Zhi X., Xu H. (2015). Synthesis of benzoxazole derivatives of honokiol as insecticidal agents against *Mythimna separata* Walker. Bioorg. Med. Chem. Lett..

[134] Singh T., Srivastava V.K., Saxena K.K., Goel S.L., Kumar A. (2006). Synthesis of new thiazolylthiazolidinylbenzothiazoles and thiazolylazetidinylbenzothiazoles as potential insecticidal, antifungal, and antibacterial agents. Arch. Pharm..

[135] Pareek D., Chaudhary M., Pareek P.K., Kant R., Ojha K.G., Iraqi S., Pareek A. (2010). Synthesis of some biologically important 2-thiobarbituric acid derivatives incorporating benzothiazole moiety. Der Pharm. Lett..

[136] Deng X.G., Guo S.X., Wang Y.M., Zhou Z.H. (2012). Synthesis and insecticidal activity of new neonicotinoid compounds. Fine Chem. Intermed..

[137] Kalavagunta P.K., Pala R., Pathipati U.R., Ravirala N. (2014). Identification of naphthol derivatives as novel antifeedants and insecticides. J. Agr. Food Chem..

[138] Li J., Wang Z.Y., Wu Q.Y., Yang G.F. (2015). Design, synthesis and insecticidal activity of novel 1, 1-dichloropropene derivatives. Pest Manag. Sci..

[139] Shang J., Li Y., Yang N., Xiong L., Wang B. (2022). Synthesis and evaluation of novel1-(((6-substitutedbenzod]thiazol-2-yl)amino)(heteroaryl) methyld) naphthalen-2-ol as pesticidal agents. J. Enzym. Inhib. Med. Ch..

